# Network pharmacological analysis and molecular docking of Huangqin-Baizhu herb pair in the treatment of threatened abortion

**DOI:** 10.1097/MD.0000000000030417

**Published:** 2022-09-09

**Authors:** Chun-xiao Dang, Ding Wang, Peng-fei Liu, Jin-xing Liu, Xiao Yu

**Affiliations:** a Shandong University of Traditional Chinese Medicine, Jinan, China; b Affiliated Hospital of Shandong University of Traditional Chinese Medicine, Jinan, China.

**Keywords:** Huangqin-Baizhu herb pair, mechanism of action, molecular docking, network pharmacology, threatened abortion, Traditional Chinese medicine

## Abstract

The incidence of threatened abortion (TA) is increasing due to poor diet and living habits, which brings great pressure to pregnant women and their families. Huangqin-Baizhu herb pair recorded in ancient books of traditional Chinese medicine has been widely used in the treatment of TA with remarkable effect. In this study, we will use the network pharmacology method to predict the target and mechanism of Huangqin-Baizhu herb pair.

**Methods:**

Traditional Chinese Medicine Systems Pharmacology Database and Analysis Platform database was used to screen the active components of Huangqin-Baizhu herb pair. Pubchem and Swiss Target Prediction databases were used to predict the action targets. Genecards, OMIM, and Drugbank databases were used to predict the related targets of TA. The intersection of drug target and disease target was selected and the intersection genes were uploaded to STRING database to construct protein–protein interaction network and conduct module analysis. Metascape database was used for Gene Ontology and Kyoto Encyclopedia of Genes and Genomes (KEGG) enrichment analysis, which was imported into Cytoscape software to construct component-pathway-gene network and finally verified by molecular docking. Ethical approval and informed consent of patients are not required because the data used in this study is publicly available and does not involve individual patient data or privacy.

**Results:**

The main active components of the herb pair are baicalein, flavanone, and norwogonin, etc. The main targets are AKT1, VEGFA, STAT3, MAPK1, SRC, etc. Cluster module analysis shows that the targets are related to cell metabolism, immune regulation and hormone level regulation. There were 2073, 3169, and 161 KEGG pathways involved in the biological processes, cell components, and molecular functions of Gene Ontology analysis, respectively. The main KEGG pathways involved in the intervention were HIF1 signaling pathway, PI3K-Akt signaling pathway, and Rap1 signaling pathway. Molecular docking showed that the main active components of the herb pair were well combined with the key targets.

**Conclusions:**

In this study, 42 active components, 152 potential targets and 11 key targets of Huangqin-Baizhu herb pair for the treatment of TA were revealed, participating in multiple signaling pathways such as PI3K-Akt, providing a theoretical basis for further experimental research.

## 1. Introduction

Threatened abortion (TA) is a small amount of vaginal bleeding with paroxysmal lower abdominal pain or low back pain before 20 weeks of gestation, and the gynecological examination shows that the cervical opening is not open and the fetal membranes are not broken. If not treated in time, it is easy to develop into inevitable or incomplete miscarriage, causing infection or even shock, which seriously threatens the life of women during pregnancy.^[1,2]^ In addition to fetal chromosomal abnormalities, the causes of TA are mostly maternal factors, such as endocrine abnormalities (luteal insufficiency, polycystic ovarian syndrome, etc), stress factors (excessive stress, anxiety, etc), or environmental factors (radiation, toxic chemicals, etc), poor lifestyle habits and trauma.^[3,4]^

Modern medicine often uses progesterone support therapy or immunotherapy, and progesterone injection and oral dydrogesterone are commonly used in clinical practice.^[5,6]^ But their application is limited due to slow absorption and poor patient compliance. TA in traditional Chinese medicine belongs to the category of “fetal leakage, fetal movement.” Kidney deficiency, Qi and blood deficiency, blood heat and blood stasis, and bruise and fall injury can affect the Chongren and uterus, making the Chongren and uterus deficient and unable to tie the fetus or the Chongren and uterus blocked, the fetus lost the nourishment, resulting in miscarriage.^[7]^ It is said that “Huangqin-Baizhu is the holy medicine to calm the fetus” from “Dan Xi Xin Fa-Jin Kui Dang Gui San Lun.” During pregnancy, due to excessive consumption of nourishing and greasy food or tonic medicine, plus little activity, the organism mostly contains internal damp-heat, so use the heat-clearing and damp-transforming medicine Huangqin-Baizhu to calm the fetus is effective. However, so far there has been no research on the mechanism of Huangqin-Baizhu herb pair in the treatment of TA.

Network pharmacology is a discipline that analyzes the drug-target-disease relationship through a network approach in conjunction with biopharmacology, data informatics and other disciplines, the holistic nature of network pharmacology is consistent with the holistic concept of Chinese medicine and the holistic nature of formulae formulation, and it is widely used to predict the active ingredients of drugs and the intersection targets with diseases, and to explore the pathways of drug treatment for diseases.^[8,9]^ This study used network pharmacology method to predict the targets and pathways of Huangqin-Baizhu herb pair for the treatment of TA in order to clarify the mechanism of action, and to better guide clinical application and experimental research.

## 2. Material and Methods

### 2.1. Collection of active ingredients and targets

We searched the keywords “Huangqin” and “Baizhu” in Traditional Chinese Medicine Systems Pharmacology Database and Analysis Platform (TCMSP, http://lsp.nwu.edu.cn/tcmsp.php), and screened them by oral bioavailability ≥ 30% and drug-likeness ≥0.18 screening of the active chemical components of Chinese medicine. Then we downloaded the Canonical SMILES structural formulae of the active chemical components through Pubchem database (https://pubchem.ncbi.nlm.nih.gov/) and imported them into Swiss Target Prediction database (http://www.swisstargetprediction.ch/) to obtain their targets.

### 2.2. Collection of disease-related targets

The keyword “threatenedabortion” wassearched in Genecards (https://www.genecards.org/), OMIM (http://www.omim.org), Drugbank (https://www.drugbank.ca/) databases to screen for disease-related targets, and the results were combined and de-duplicated. Then we imported the targets of the Huangqin-Baizhu herb pair and the related targets of TA into the BMK cloud platform to obtain their intersection target genes, which were the potential targets of Huangqin-Baizhu herb pair for the treatment of TA.

### 2.3. Construction of protein–protein interaction network

We imported the potential targets of Huangqin-Baizhu herb pair for the treatment of TA into the STRING database (https://string-db.org/cgi/input.pl) to construct the protein–protein interaction (PPI) network and imported the results into Cytoscape 3.8.0 software to better visualize the protein interactions. Then, we used Network analyzer plug-in to regulate the mapping network and screen out the core proteins by Network topology analysis, while the intersection genes were analyzed by Cluster module.

### 2.4. GO, KEGG enrichment analysis, and visualization network

The potential targets of Huangqin-Baizhu herb pair for the treatment of TA were imported into the Metascape database (http://metascape.org/) for Gene Ontology (GO) and Kyoto Encyclopedia of Genes and Genomes (KEGG) enrichment analysis to explore the key signaling pathways of Huangqin-Baizhu herb pair for the treatment of TA. We selected the top 20 pathways and their associated targets in descending order of *P* value and imported them into Cytoscape 3.8.0 software to construct a component-pathway-gene network and performed network topology analysis.

### 2.5. Molecular docking analysis

The sdf format files of the Chinese medicine components were downloaded from ZINC database (http://zinc.docking.org/), and the pdb format files of the 2D structure of the target protein were downloaded from PDB database (https://www.rcsb.org/), and the Discovery Studio software was used to dehydrate, hydrogenate. Finally, we performed LibDock analysis on the Chinese herb components and the treated target proteins, and also imported PyRx software for VINA docking, the smaller the binding energy indicated the stronger the binding power.

## 3. Results

### 3.1. Active components and targets of TCM

Fifty-five kinds of active components of Baizhu and 143 kinds of Huangqin were obtained by TCMSP database, and 7 kinds of active components of Baizhu and 35 kinds of Huangqin were further screened by oral bioavailability ≥ 30% and drug-likeness ≥ 0.18 (Table 1). The target genes were searched by Pubchem database and Swiss Target Prediction database, and a total of 544 target genes were obtained from the Huangqin-Baizhu herb pair after combining and de-duplicating.

**Table 1:** The active ingredient of Huangqin-Baizhu herb pair.

Mol ID	Molecule Name	OB (%)	DL	Score
MOL001689	Acacetin	34.97	0.24	Huangqin
MOL000173	Wogonin	30.68	0.23	Huangqin
MOL000228	(2R)-7-hydroxy-5-methoxy-2-phenylchroman-4-one	55.23	0.2	Huangqin
MOL002714	Baicalein	33.52	0.21	Huangqin
MOL002908	5,8,2′-Trihydroxy-7-methoxyflavone	37.01	0.27	Huangqin
MOL002909	5,7,2,5-tetrahydroxy-8,6-dimethoxyflavone	33.82	0.45	Huangqin
MOL002910	Carthamidin	41.15	0.24	Huangqin
MOL002911	2,6,2′,4′-tetrahydroxy-6′-methoxychaleone	69.04	0.22	Huangqin
MOL002913	Dihydrobaicalin_qt	40.04	0.21	Huangqin
MOL002914	Eriodyctiol (flavanone)	41.35	0.24	Huangqin
MOL002915	Salvigenin	49.07	0.33	Huangqin
MOL002917	5,2′,6′-Trihydroxy-7,8-dimethoxyflavone	45.05	0.33	Huangqin
MOL002925	5,7,2′,6′-Tetrahydroxyflavone	37.01	0.24	Huangqin
MOL002926	Dihydrooroxylin A	38.72	0.23	Huangqin
MOL002927	Skullcapflavone II	69.51	0.44	Huangqin
MOL002928	Oroxylin a	41.37	0.23	Huangqin
MOL002932	Panicolin	76.26	0.29	Huangqin
MOL002933	5,7,4′-Trihydroxy-8-methoxyflavone	36.56	0.27	Huangqin
MOL002934	NEOBAICALEIN	104.34	0.44	Huangqin
MOL002937	DIHYDROOROXYLIN	66.06	0.23	Huangqin
MOL000358	Beta-sitosterol	36.91	0.75	Huangqin
MOL000359	Sitosterol	36.91	0.75	Huangqin
MOL000525	Norwogonin	39.4	0.21	Huangqin
MOL000552	5,2′-Dihydroxy-6,7,8-trimethoxyflavone	31.71	0.35	Huangqin
MOL000073	Ent-Epicatechin	48.96	0.24	Huangqin
MOL000449	Stigmasterol	43.83	0.76	Huangqin
MOL001458	Coptisine	30.67	0.86	Huangqin
MOL001490	bis[(2S)-2-ethylhexyl] benzene-1,2-dicarboxylate	43.59	0.35	Huangqin
MOL001506	Supraene	33.55	0.42	Huangqin
MOL002879	Diop	43.59	0.39	Huangqin
MOL002897	Epiberberine	43.09	0.78	Huangqin
MOL008206	Moslosooflavone	44.09	0.25	Huangqin
MOL010415	11,13-Eicosadienoic acid, methyl ester	39.28	0.23	Huangqin
MOL012245	5,7,4′-trihydroxy-6-methoxyflavanone	36.63	0.27	Huangqin
MOL012246	5,7,4′-trihydroxy-8-methoxyflavanone	74.24	0.26	Huangqin
MOL012266	Rivularin	37.94	0.37	Huangqin
MOL000020	12-senecioyl-2E,8E,10E-atractylentriol	62.4	0.22	Baizhu
MOL000021	14-acetyl-12-senecioyl-2E,8E,10E-atractylentriol	60.31	0.31	Baizhu
MOL000022	14-acetyl-12-senecioyl-2E,8Z,10E-atractylentriol	63.37	0.3	Baizhu
MOL000028	α-Amyrin	39.51	0.76	Baizhu
MOL000033	(3S,8S,9S,10R,13R,14S,17R)-10,13-dimethyl-17-[(2R,5S)-5-propan-2-yloctan-2-yl]-2,3,4,7,8,9,11,12,14,15,16,17-dodecahydro-1H-cyclopenta[a]phenanthren-3-ol	36.23	0.78	Baizhu
MOL000049	3β-acetoxyatractylone	54.07	0.22	Baizhu
MOL000072	8β-ethoxy atractylenolide III	35.95	0.21	Baizhu

DL = drug-likeness, OB = oral bioavailability.

### 3.2. Disease targets and intersection genes

**Figure 1.**
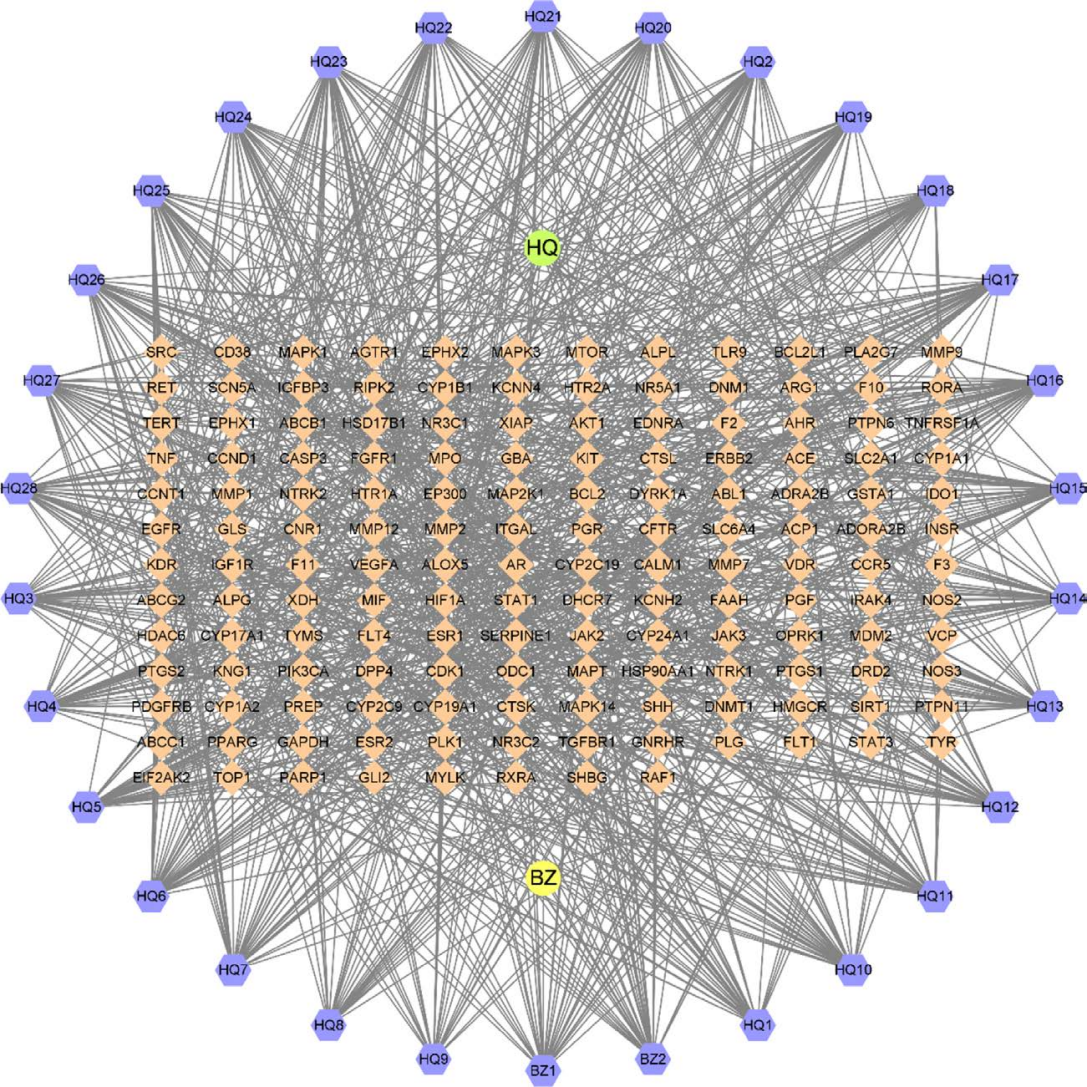
“Drug-component-gene” network.

We searched Genecards, OMIM, and Drugbank databases for disease targets using the keyword “TA,” and the results were combined and de-duplicated to obtain 1071 target genes for TA. After the intersection of the targets of Huangqin-Baizhu herb pair and the disease targets, 152 common targets were obtained and imported into Cytoscape 3.8.0 software to construct a drug-component-gene network (Fig. 1). According to the number of target genes of active components, the top 5 active components of Huangqin-Baizhu herb pair were found to be baicalein, flavanone, norwogonin, 5,7,4′-trihydroxy-8-methoxyflavone and acacetin, as shown in Table 2.

**Table 2:** Top 5 active compounds with the most targets.

Number	Mol ID	Molecule Name	OB (%)	DL	Source	Number of targets
1	MOL002714	Baicalein	33.52	0.21	Huangqin	39
2	MOL002914	Flavanone	41.35	0.24	Huangqin	39
3	MOL000525	Norwogonin	39.40	0.21	Huangqin	39
4	MOL002933	5,7,4′-Trihydroxy-8-methoxyflavone	36.56	0.27	Huangqin	38
5	MOL001689	Acacetin	34.97	0.24	Huangqin	37

DL = drug-likeness, OB = oral bioavailability.

### 3.3. PPI core network

**Figure 2.**
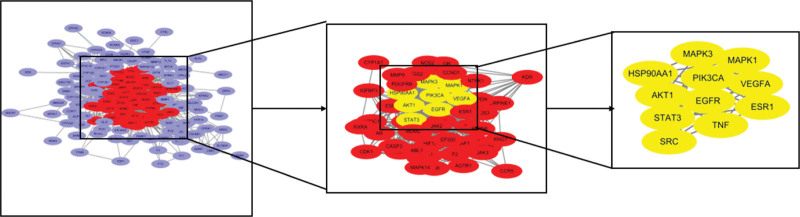
PPI network core (CytoNCA).

The 152 intersection genes obtained in 2.2 were imported into the STRING database, setting the minimum interaction score to 0.7 and hiding the free nodes, the results showed that a total of 152 targets were interlinked, the network had 859 edges with an average node degree of 11.3 and an average clustering coefficient of 0.488. Downloaded its TSV file and imported it into Cytoscape 3.8.0 software. Through network topology analysis, screened twice with the median of degree value as the lower limit, further explored the key target genes, and obtained the key target genes of Huangqin-Baizhu herb pair for the treatment of TA: AKT1 (degree: 52), VEGFA (degree: 51), STAT3 (degree: 51), MAPK1 (degree: 45), SRC (degree: 42), MAPK3 (degree: 39), HSP90AA1 (degree: 38), PIK3CA (degree: 37), EGFR (degree: 37), TNF (degree: 34) and ESR1 (degree: 33) (Fig. 2).

### 3.4. Cluster module analysis

**Figure 3.**
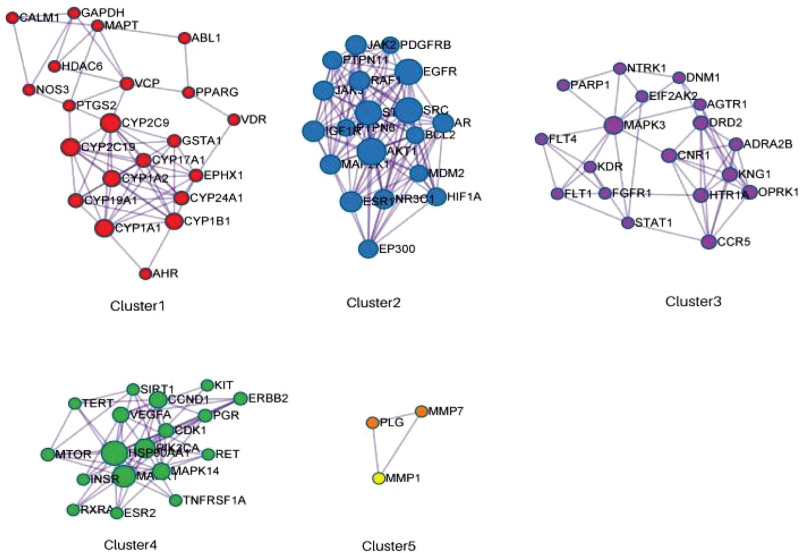
Cluster module analysis.

Cluster module analysis of the intersection genes yielded a total of 5 modules (Fig. 3), and GO enrichment analysis of the 5 modules revealed that Cluster1 was mainly related to heterologous metabolism, olefin metabolism, and response to foreign body stimulation; Cluster2 was mainly associated with regulation of hormone levels and the protein tyrosine kinase signaling pathway; Cluster3 was mainly regulated through the regulation of kinases, transferases and protein phosphorylation; Cluster4 was mainly related to the regulation of MAPK cascade; Cluster5 mainly affected the extracellular matrix and structural organization.

### 3.5. GO, KEGG enrichment analysis, and visualization network

**Figure 4.**
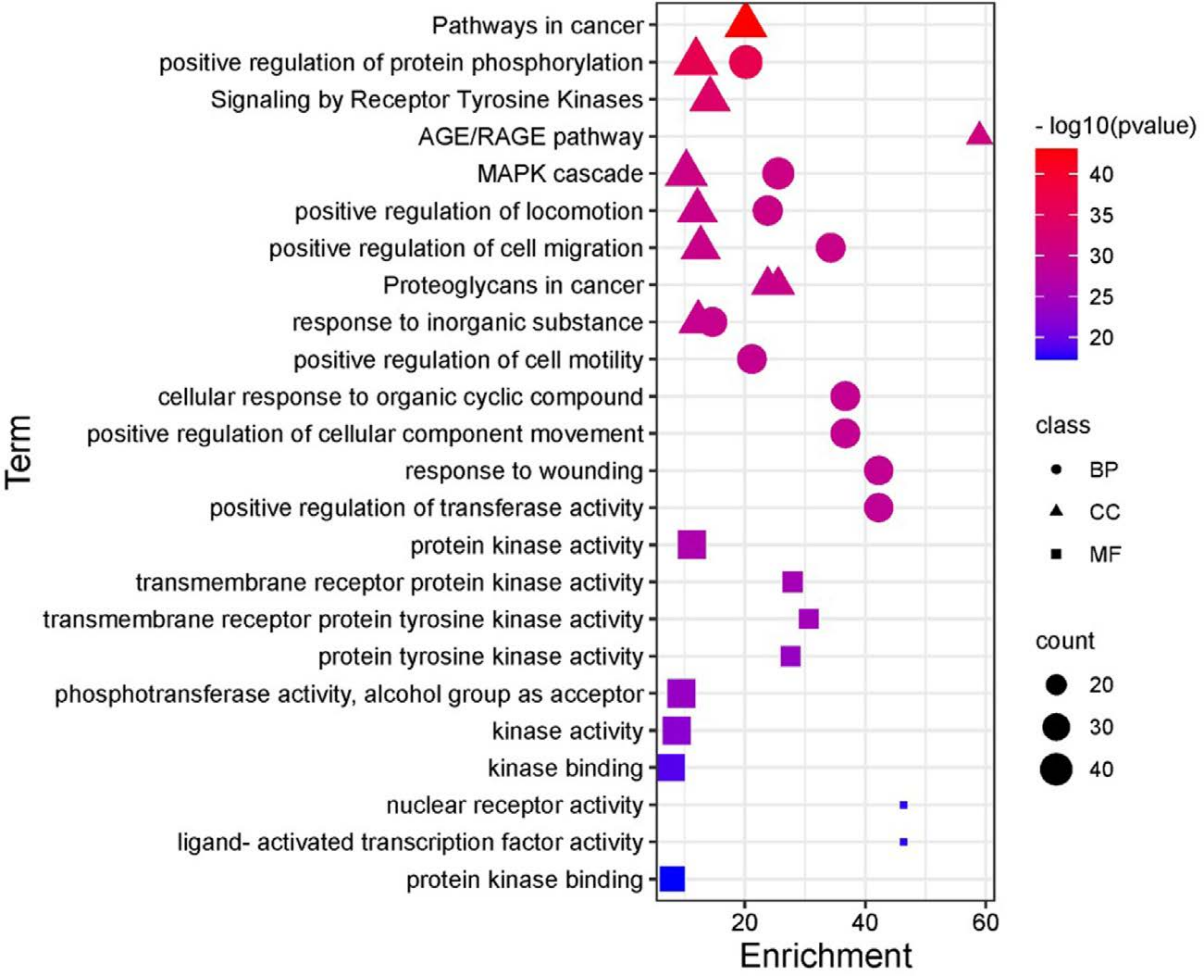
Bubble Diagram of signal pathway enrichment of key targets.

**Figure 5.**
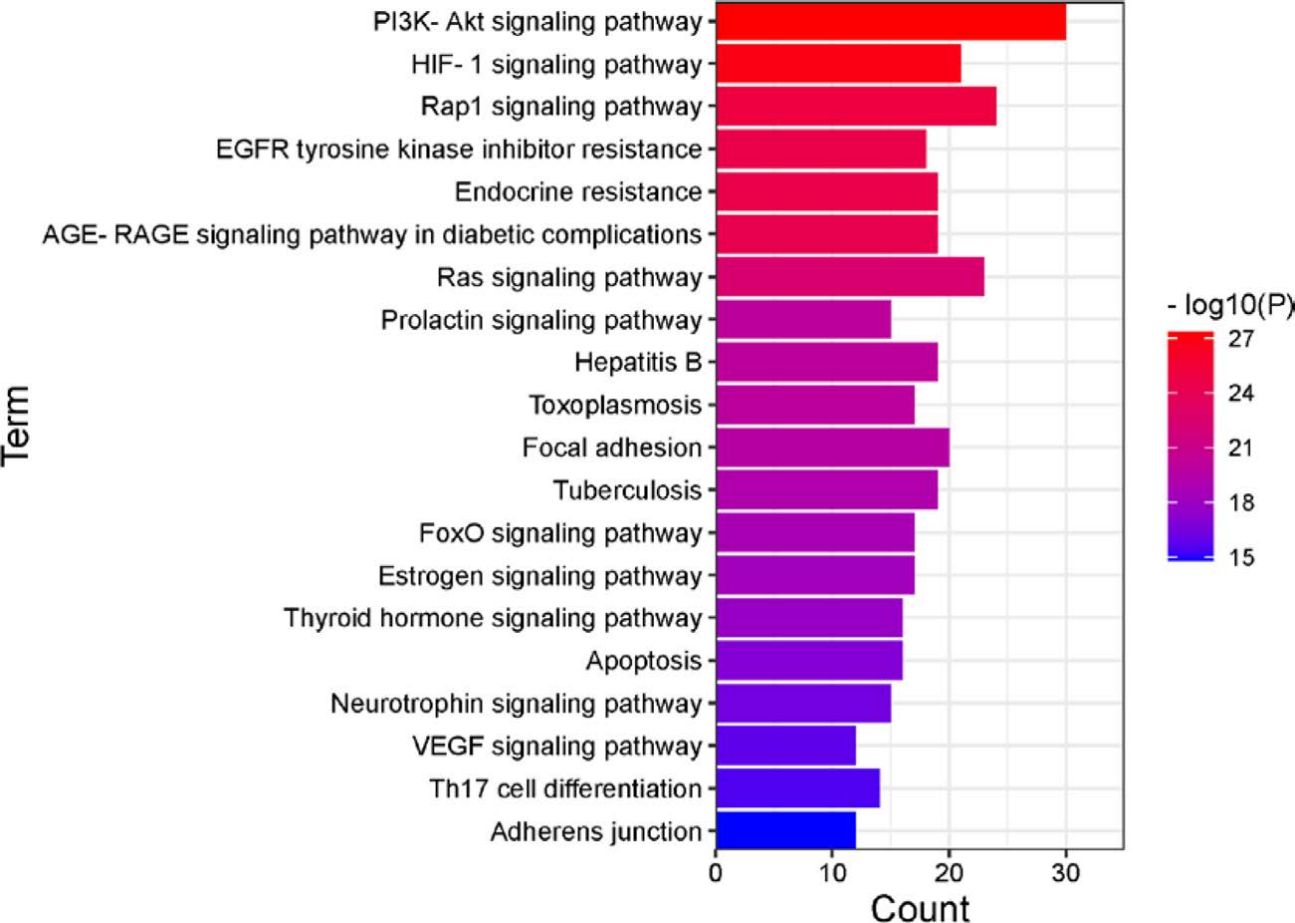
Barplot histogram of signal pathway of key target enrichment.

**Figure 6.**
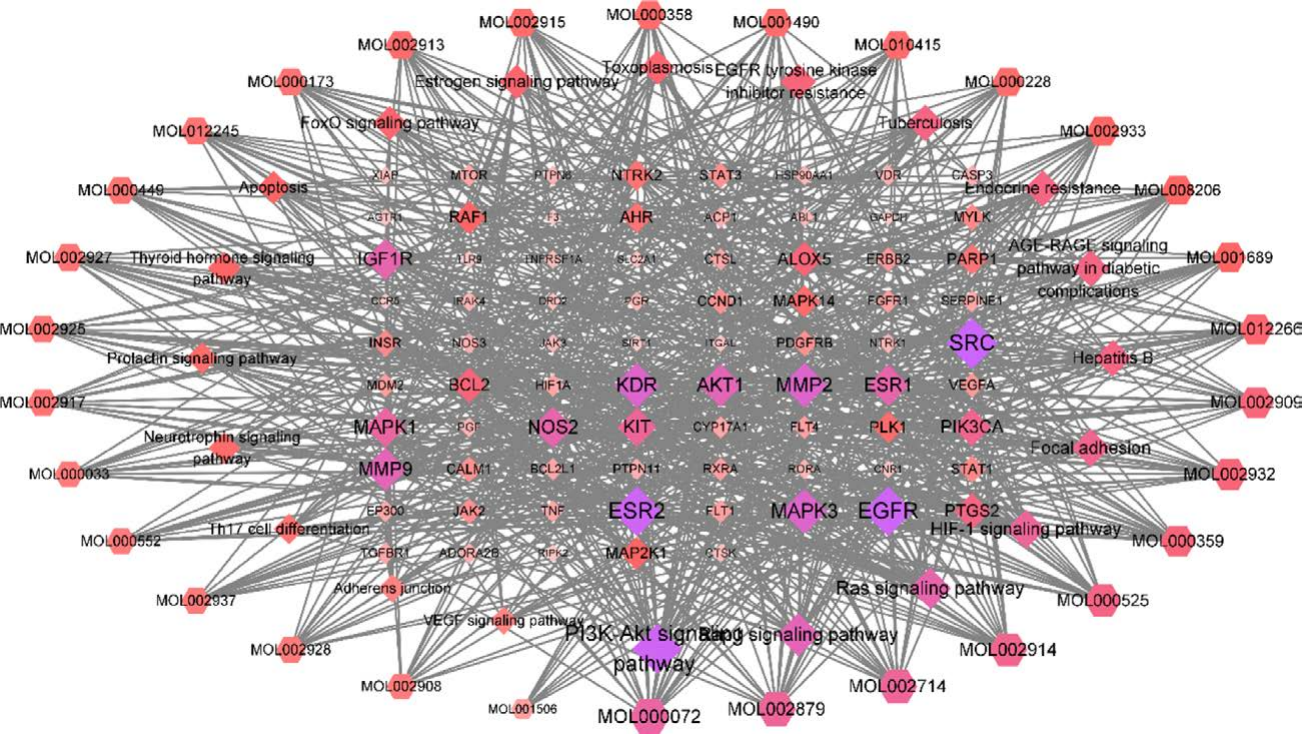
“Component-pathway-gene” network.

The 152 intersection genes were imported into the Metascape database for enrichment analysis, and a total of 5403 GO entries were obtained, including 2073 for biological processes, 3169 for cellular components, and 161 for molecular functions. After sequencing according to *P* value from small to large, it was found that Huangqin-Baizhu herb pair mainly participated in regulating protein phosphorylation, MAPK cascade and cell proliferation and migration through cell metabolism, protein phosphorylation and receptor tyrosine kinase signal transduction, and the target mainly included protein kinase and receptor protein tyrosine kinase. KEGG analysis showed that there were 294 pathways, and after screening out irrelevant pathways, it was found that it was most closely related to HIF1 signaling pathway (degree: 22), PI3K-Akt signaling pathway (degree: 30), Rap1 signaling pathway (degree: 24). Specifically, see Figures 4 and 5, the darker the color, the smaller the *P* value, the stronger the significance, the larger the icon area, the more target genes involved in this process. The component-pathway-gene network showed that the number of target genes corresponding to the PI3K-Akt signaling pathway and 8β-ethoxylolactone III was the largest, as shown in Figure 6.

### 3.6. Molecular docking results

**Figure 7.**
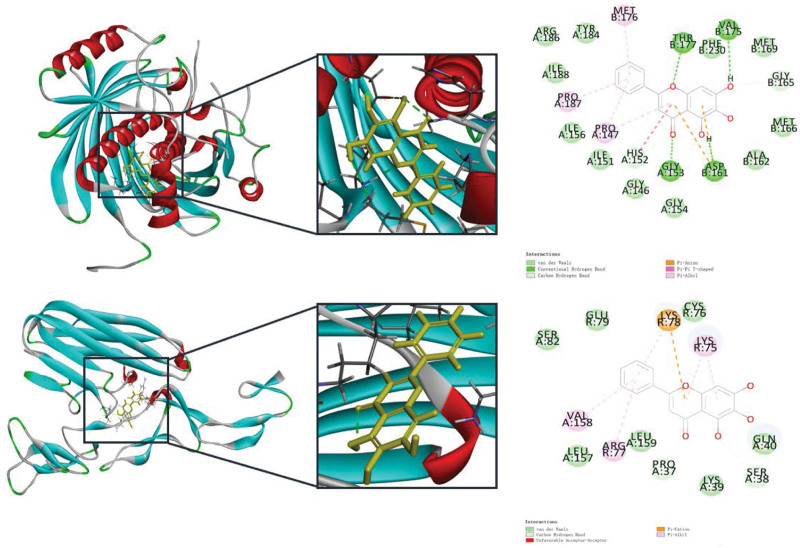
Interaction of baicalein with targets.

The molecular docking of baicalein, the main component of the drug pair, with the top 11 core target genes showed that the binding energy of baicalein was lower than −4.52 kcal/mol with each target gene, among which the binding energy with 8 target genes was lower than −7.00 kcal/mol. The binding energy lower than −4.52 indicated a certain binding ability between the 2, and lower than 7.00 indicated a stronger binding power between the 2. The LibDock analysis showed that the scores were higher than 90 except for MAPK1, which indicated that the binding between the subject and the ligand was better, and the higher the score, the more stable the structure of the resulting complex (Table 3). The AKT1 and TNF target genes with better binding and complex stability among 11 core target genes of baicalin were selected for visualization and analysis. The results showed that baicalein formed a hydrogen bond with amino acid residues VALB-175, ASPB-161, THRB-177, GLYA-153 of AKT1, 2 salt bridges with ASPB-161, and amino acid residues SERA-38 of TNF by hydrogen bonding, and a salt bridge with LYSR-78, as shown in Figure 7.

**Table 3:** Binding energy of baicalein to the top 11 targets.

Target	PDB ID	The binding energy/(kcalmol^-1^)	LibDock Score
AKT1	4GAH	−9.5	123.09
VEGFA	6V7K	−6.5	92.42
STAT3	6QHD	−8.7	97.10
MAPK1	6G54	−8.1	78.04
SRC	3D7T	−8.7	94.39
MAPK3	6GES	−8.5	96.62
HSP90AA1	5NJX	−7.0	90.78
PIK3CA	6GVH	−8.6	104.52
EGFR	3IKA	−8.3	98.52
TNF	1TNR	−6.7	108.08
ESR1	6KN5	−7.6	95.30

## 4. Discussion

With the rapid development of modern society, women are facing pressure from family, society and other aspects, the incidence of TA has increased to 20%,^[10]^ and bed rest during pregnancy to protect the fetus plus excessive consumption of nourishing and greasy products, so that the body is often in a state of internal damp-heat. Although the etiology of TA is clearer in modern medicine, the treatment is limited.^[11]^ And Chinese medicine for fetal preservation, as a characteristic advantage of TCM gynecology, with high success rate of improving pregnancy outcome and low adverse effects, should be the focus of future research. Huangqin-Baizhu herb pair, as high-frequency drugs in TCM for the treatment of TA and habitual abortion,^[12,13]^ are widely used and have significant efficacy because their functions are to clear heat and dry dampness, strengthen the spleen, benefit qi, and calm the fetus, which are more suitable for modern people’s physique and living habits. In this study, by constructing the drug-component-gene network, we predicted that there were 544 target genes of Huangqin-Baizhu herb pair, including 162 target genes for the treatment of TA, and then by constructing the PPI network, we found that the key target genes of Huangqin-Baizhu herb pair for the treatment of TA were AKT1, VEGFA, STAT3, etc. Further enrichment analysis of the target genes showed that the Huangqin-Baizhu herb pair mainly treated TA through HIF1 signaling pathway, PI3K-Akt signaling pathway and Rap1 signaling pathway. Molecular docking also showed that the components of the herb pair were well combined with the target genes.

Baicalein, flavanone, norwogonin, 5,7,4′-trihydroxy-8-methoxyflavone, and acacetin are the main active ingredients of Huangqin-Baizhu herb pair for the treatment of TA. Baicalein and norwogonin can enhance the immunomodulatory function of the body,^[14,15]^ and baicalein can dilate blood vessels and improve uterine blood circulation to nourish the embryo while inhibiting allergic and metabolic reactions, and also has a wide range of antibacterial effects.^[16]^ Clinical studies^[17,18]^ have shown that an appropriate amount of TNF-α in pregnant women can promote the secretion of progesterone and human chorionic gonadotropin, guarantee the maternal energy supply and fetal development, maintain pregnancy and initiate delivery. However, high levels of TNF-α in women with TA can cause miscarriage or premature birth. The active ingredients such as 5,7,4′-trihydroxy-8-methyldihydroflavone, baicalein and norwogonin can inhibit the production of TNF-α by T cells and NK cells and promote the production of cytokines such as IL-6 and IL-8 by Th2 cells to maintain pregnancy.^[19,22]^ Acacetin, flavanone, and 5,7,4′-trihydroxy-8-methoxyflavone can inhibit the production of TNF-α through MAPKs signaling pathway to inhibit protein kinase and protein tyrosine kinase activity, thus reducing the histamine release reaction process in the body to achieve bactericidal and anti-inflammatory effects,^[20–22]^ maintaining the uterine cavity environment and reducing the abortion rate. In addition, many animal experiments have reported that acacetin, in addition to its antibacterial effect,^[23]^ can also reduce the expression of Kv1.3 protein and the activation of immune T cells, thereby inhibiting inflammation-mediated autoimmune diseases.^[24]^

VEGFA, a member of the VEGF family, can bind to 3 tyrosine kinase receptors, VEGFR1, VEGFR2, and VEGFR3, causing them to autophosphorylate.^[25]^ Thus it can bind to the p85 subunit of PI3K and phospholipase C-γ (PLCγ), which in turn transmits information through the PKB/AKT signaling pathway to increase vascular permeability and promote cell proliferation and migration, thereby promoting embryonic growth and development.^[26,27]^ AKT is a serine/threonine protein kinase, and AKT1, a heterodimer of AKT, mediates many PI3K-regulated downstream pathways.^[28]^ Some studies have found^[29,30]^ that AKT phosphorylation can also be involved in blood vessel formation through the PKB/AKT pathway. The levels of PI3K and AKT in the chorionic villus cells of spontaneously aborted patients are lower than those of artificially aborted patients, suggesting that the PI3K/AKT pathway can promote the growth of trophoblast cells and regulate the function of chorionic trophoblast cells, suggesting that the PI3K/AKT pathway has direct and indirect effects on embryonic development and pregnancy maintenance.^[31]^ As the research progressed, it was found that STAT3 pathway expression was inhibited, its downstream regulatory protein Cyclin D1 expression was decreased, ovarian granulosa cell activity was stopped and proliferation was inhibited,^[32]^ thus affecting the hormone level of the body. Relevant clinical experiments^[33]^ have also confirmed that the expression of Cyclin D1 in chorionic villus cells and decidual cells of spontaneous abortion patients is lower than that of normal pregnancy patients, and the number of trophoblast cells decreased and cell apoptosis increased. The inhibition of STAT3 also affects the expression of VEGF and the differentiation of Th17 cells, and the levels of related cytokines TNF-α, IL-6, IL-8, and IL-17 decrease,^[34]^ resulting in immune imbalance and reduced angiogenesis, which affects embryonic development. Endometrial hypoxia-inducible factor HIF exerts its biological activity as a heterodimeric protein by the polymerization of HIF-α and HIF-β to form HIF1 protein.^[35]^ HIF1 protein is expressed in both miscarried and normal pregnancies because the development of the embryo is completed in a relatively low oxygen environment in early pregnancy. However, HIF1-α protein overexpression in early miscarriage causes endometrium to be infiltrated by inflammatory cells and its receptivity to the embryo to be reduced. Meanwhile, gestation trophoblast cells are difficult to invade the uterine spiral artery and placental blood perfusion is reduced.^[35,36]^ Other studies have found that HIF1-α levels are proportional to microvascular density MVD, and decreased MVD levels can lead to insufficient placental angiogenesis and embryonic ischemia and hypoxia for survival,^[37]^ and there may be a complex bidirectional relationship between the HIF pathway and the maintenance of pregnancy. RAS-associated protein 1 (RAP1) is composed of RAP1A, RAP1B two heterodimers, FOXO3 can resist the damage of HIF1-α, and also promotes methylation of the endometrium and placenta formation.^[38,39]^ It was found that RAP1 can activate the FOXO3 pathway,^[40]^ and also promote the growth of ovarian tissue, increase the level of E_2_ and P in the body through the MAPK pathway. And in vitro and in vivo experiments have found that RAP1 acts on the AKT signaling pathway to promote the growth of trophoblast cells,^[41]^ ensuring that the early embryo receives sufficient trophoblast. In addition, clinical and laboratory experiments^[42,43]^ have found that the levels of RAP1 and EPAC1 are higher in ectopic endometrium than in normal endometrium and they are positively correlated. It is speculated that RAP1 and EPAC1 may play a synergistic role in promoting the formation of new microvessels in endometrium and the proliferation and adhesion of endometrial cells, which are beneficial to embryo implantation.

## 5. Conclusions

This study revealed 42 active components, 152 potential target genes and 11 key target genes of Huangqin-Baizhu herb pair for the treatment of TA, which are involved in various signaling pathways such as PI3K-Akt. The main effects of Huangqin-Baizhu herb pair on the treatment of TA are to promote the proliferation and differentiation of endometrial and trophoblastic cells, the formation of endometrial microvasculature and placenta, and to maintain the stability of the body’s immune environment to maintain pregnancy and prevent miscarriage. At present, there are few experimental studies on the effect of Huangqin-Baizhu herb pair on the treatment of TA, and this study provides a theoretical analysis and prediction of the mechanism, which can provide a theoretical basis for future studies. However, there are a number of issues that have not yet been determined, such as identifying the relative content of active constituents in the herb pair and interpreting the pharmacological effects when multiple target genes are activated. Therefore, more in vitro and in vivo experiments are needed for further exploration. In addition, with the continuous updating of drug and disease target gene databases and the development of medical research, the mechanisms and pathways of Huangqin-Baizhu herb pair for the treatment of TA will be further expanded, and their clinical value needs to be further explored. How to rationally use TCM and combine it with modern medicine to treat diseases should be the focus of future research.

## Acknowledgment

We thank all the authors for their full cooperation, for the information, literature search and collection, and for the strong guidance on the knowledge and logical order of the subject matter, without which this manuscript would not have been completed. Thanks to the National Natural Science Foundation of China and the Natural Science Foundation of Shandong Province for their sponsorship.

## Author contributions

Conceptualization: C.D., J.L.; Data curation: C.D., D.W., X.Y.; Formal analysis: C.D., D.W.; Funding acquisition: P.L., J.L., X.Y.; Investigation: C.D., D.W., X.Y.; Methodology: C.D., D.W.; Writing – original draft: C.D.; Writing – review & editing: P.L., J.L.
